# *Agrimonia procera* exerts antimicrobial effects, modulates the expression of defensins and cytokines in colonocytes and increases the immune response in lipopolysaccharide-challenged piglets

**DOI:** 10.1186/s12917-018-1680-0

**Published:** 2018-11-15

**Authors:** Tobias Gräber, Holger Kluge, Sebastian Granica, Gert Horn, Jutta Kalbitz, Corinna Brandsch, Antje Breitenstein, Christine Brütting, Gabriele I. Stangl

**Affiliations:** 1 Institute of Agricultural and Nutritional Sciences, Martin Luther University Halle-Wittenberg, Von-Danckelmann-Platz 2, 06120 Halle (Saale), Germany; 2 Department of Pharmacognosy and Molecular Basis of Phytotherapy, Faculty of Pharmacy, Medical University of Warsaw, Banacha St. 1, 02-097 Warsaw, Poland; 3 Exsemine GmbH, Am Wehr 4, 06198 Salzatal, Germany; 4 BioSolutions Halle GmbH, Weinbergweg 22, 06120 Halle (Saale), Germany

**Keywords:** *Agrimonia procera*, Agrimoniin, Caco-2, Cytokine expression, Growth performance, Lipopolysaccharides (LPS), Pig, TNFα

## Abstract

**Background:**

Because antibiotic use in livestock is assumed to contribute to the emerging public health crisis of antibiotic resistance, alternatives are required. Phytogenic additives are extensively studied due to their antibiotic properties. Components of *Agrimonia* species have been reported as candidate antimicrobials that possess antioxidative and anti-inflammatory properties. We studied the impact of *Agrimonia procera* (AP) on the growth of selected strains of gut bacteria, the effect of AP on the mRNA abundance of genes involved in inflammation and bacterial defense in a colon carcinoma cell line, the effect of AP in piglets challenged with lipopolysaccharides, and the effect of AP on the growth performance of healthy piglets.

**Results:**

The in vitro growth rate of different bacteria strains was negatively affected by AP, especially in *Pediococcus pentosaceus* and all tested *E. coli* strains. Stimulation of Caco-2 cells with TNFα resulted in elevated mRNA expression of CXCL1, IL-8 and GPX2. After pretreatment of cells with AP, stimulation of Caco-2 cells with TNFα still resulted in elevated mRNA expression of CXCL1 and IL-8 at all measured points in time. However, mRNA expression in AP-pretreated cells was lower after 6 h and 24 h. In addition, expression of DEFB1 and GPX2 was significantly elevated after TNFα stimulation. In vivo, application of lipopolysaccharides induced significantly increased animal body temperatures. Piglets pretreated with AP prior to lipopolysaccharide application showed a faster and larger increase in body temperature than controls. In addition, piglets pretreated with AP appeared to release more TNFα than controls. In healthy piglets, AP treatment had no impact on growth performance parameters. Fecal dry matter and total plasma antioxidant capacity tended to be higher in piglets treated with AP than in control piglets (*P* = 0.055 and *P* = 0.087, respectively).

**Conclusions:**

AP has antimicrobial effects in vitro and stimulated the expression of proinflammatory cytokines in Caco-2 cells. The additive had no effect on growth in healthy piglets but increased the immune response in LPS-treated animals. In addition, AP appeared to have antioxidative effects in vivo. Therefore, AP merits testing as a future alternative to antibiotics in animal husbandry.

## Background

Diseases affecting livestock are normally associated with a negative impact on animal productivity. Maintenance of general health and prevention of infectious diseases are critically dependent on intestinal homeostasis and proper immune competence. Early in life, stress factors such as the hygiene status of the postweaning room, air quality, group size and other husbandry conditions [1], as well as the composition of the gut microflora [2], are critical factors that influence the susceptibility of pigs to gastrointestinal infections. Antibiotics are used to treat ill animals or a batch of animals when at least one is diagnosed with a bacterial infection. Because the use of antibiotics in livestock animals is suggested to play a major role in the emerging public health crisis of antibiotic resistance [3], alternatives are required. In an attempt to improve animal welfare, phytogenic additives are being extensively studied as an alternative to antibiotics due to their antibiotic properties [4, 5]. Polyphenols or polyphenol-rich extracts derived from forage crops or medicinal plants also appear to be able to reduce or inhibit inflammatory processes in cells and experimental animals [6–11].

Components of *Agrimonia* species, members of the family Rosaceae, have been reported as candidate antibiotic feed additives that possess antioxidative and anti-inflammatory properties. Zhu et al. [12] identified five flavonoids from *Agrimonia pilosa* Ledeb. – taxifolin, catechin, hyperoxide, quercitrin and rutin – that are particularly effective against oxidative DNA damage. Recent data have shown that the polyphenolic fraction of *Agrimonia eupatoria L.* exerts anti-inflammatory activity in LPS-stimulated macrophages and in a mouse model of carrageenan-induced paw edema [13]. In humans, the consumption of tea made of *A. eupatoria* L. significantly elevated the total antioxidant capacity of plasma and significantly lowered the level of interleukin-6 [14]. Seeds from *A. eupatoria* have been reported as candidates for antibiotic use owing to their antibacterial properties [15]. This is the reason why traditional medicine uses *A. eupatoria* and other *Agrimonia* species for treatment of diarrhea [16]. In Europe, one of the most common *Agrimonia* species is *Agrimonia procera* Wallr. (AP). The major bioactive compounds found in this species are polyphenols such as flavonoids, mainly glycosides of luteolin and apigenin. AP also contains larger amounts of the bitter-tasting compound agrimoniin [17]. Recently, we were able to show that treatment of porcine peripheral blood monocytes with AP extract reduced the mRNA abundance of TNFα in cells challenged with LPS but not in unchallenged cells [18]. However, the available data do not allow a final evaluation of whether AP and its bioactive compounds could be used for the prevention and treatment of infectious diseases in pigs.

Therefore, the current studies aimed to elucidate (i) the impact of AP on the growth of selected strains of gut bacteria; (ii) the effect of AP on the mRNA abundance of genes involved in inflammation, bacterial defense and radical scavenging in a colon carcinoma cell line; (iii) the effect of AP in pigs challenged with LPS; and (iv) the effect of AP on the growth performance of healthy pigs.

## Results

### Characterization of secondary plant compounds in *Agrimonia procera*

The most prominent polyphenol in AP samples was agrimoniin, with a concentration of 27.9 mg per g of dry matter (Table 1). The flavones apigenin and luteolin were found as glycosides and glucuronides, with a higher concentration of 7-O-glucuronides than of 7-O-glycosides (Table 1). The flavonol quercetin was detected only as glycosidic compound (quercitrin). Kaempferol and procyanidins could not be detected in AP.

**Table 1:** Analyzed content of agrimoniin and related polyphenols in *Agrimonia procera* plant (mean; *n* = 2)

	mg/g dry matter
Agrimoniin	27.9
Apigenin 7-O-glucuronide	2.18
Luteolin-7-O-glucuronide	1.46
Hyperoside	0.38
Apigenin 7-O-glucoside	0.21
Luteolin-7-O-glucoside	0.17
Quercitrin	0.04

### Antimicrobial efficacy test of *Agrimonia procera*

**Fig. 1:**
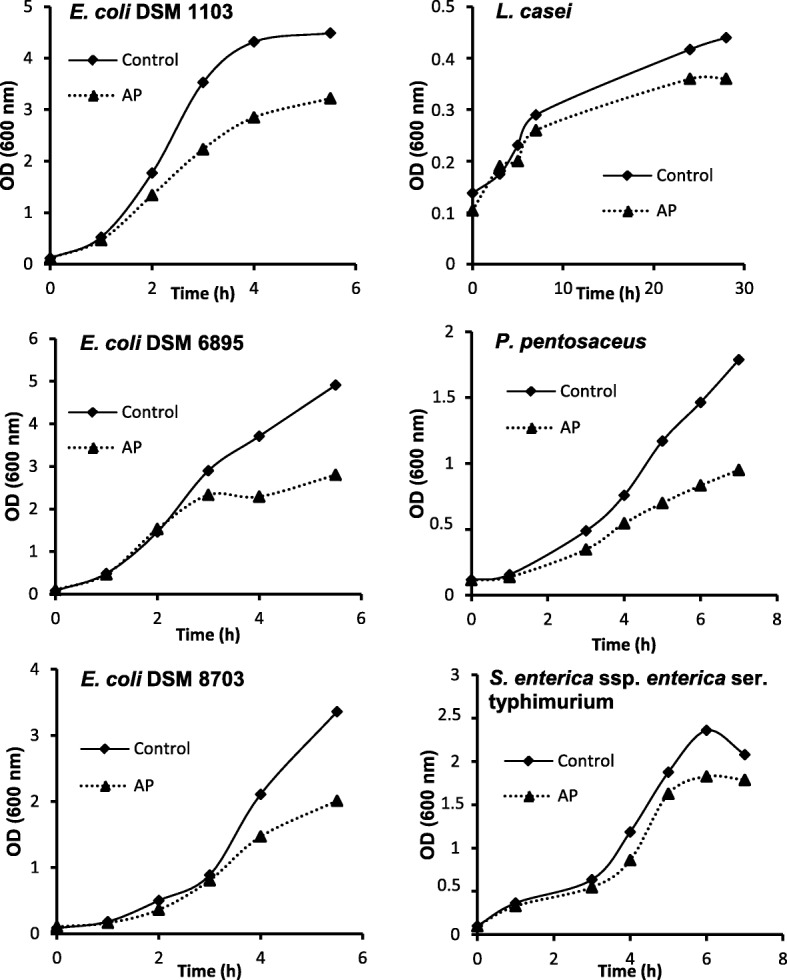
Growth of bacterial strains with and without *Agrimonia procera* (AP). Growth of *E. coli* DSM 6895, *E. coli* DSM 8703, *E. coli* DSM 1103, *Lactobacillus casei*, *Pediococcus pentosaceus* and *Salmonella enterica* ssp*. enterica* serotype *Typhimurium* analyzed by assessing the optical density at 600 nm over a period of up to 28 h. Different periods of examination are caused by different doubling times of bacterial strains. Bacterial strains were cultivated in medium at 37 °C with aqueous extracts of *Agrimonia procera* (AP, 1 mg/ml). Medium without AP was used for the controls

The growth kinetics curves of *E. coli* DSM 6895, *E. coli* DSM 8703, *E. coli* DSM 1103, *Lactobacillus casei*, *Pediococcus pentosaceus* and *Salmonella enterica* ssp*. enterica* serotype Typhimurium, as measured by turbidity at 600 nm, are shown in Fig. 1. After 3 h, growth inhibition was apparent in most of the AP-treated strains. After 5 h 30 min, the growth rates of AP-treated *E. coli* strains and *P. pentosaceus* decreased to 57, 60 and 72% compared to the control. The growth inhibition of AP-treated *Salmonella* was considerably slower than that of *E. coli*.

The maximum effect of AP on the growth of *Salmonella* was observed after 6 h (− 23%). In contrast, the growth rate of *L. casei* was hardly affected by AP.

### In vitro experiments with Caco-2 cells

**Fig. 2:**
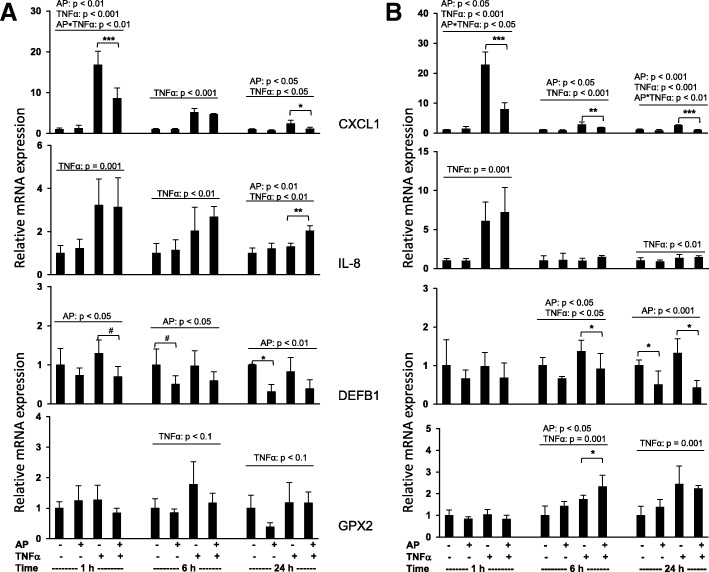
Relative mRNA expression of defensins and cytokines in Caco-2 cells. Relative mRNA expression of chemokine (C-X-C motif) ligand (CXCL1), interleukin-8 (IL-8), beta-defensin 1 (DEFB1) and glutathione peroxidase 2 (GPX2) in Caco-2 cells **a** without agrimoniin (AP) pretreatment and **b** with AP pretreatment for 4 h. Cells were coincubated with or without TNFα (10 ng/ml) and with or without AP (50 μM) for 1 h, 6 h and 24 h. Data represent the mean values ± SD (1 h, 6 h, *n* = 4; 24 h: *n* = 3). Data were analyzed by two-way ANOVA with the classification factors AP and TNFα and the interaction between those two factors. Individual means at any given point in time were compared by Fisher’s exact test. Differences between AP-treated (+) and non-AP-treated (−) cells are indicated by asterisks: ^#^*p* < 0.1; *p < 0.05; **p < 0.01; ***p < 0.001

In the first experiment, we investigated the role of AP on gene expression in TNFα-treated and untreated cells of the human colon carcinoma cell line Caco-2 (Fig. 2a). The second experiment aimed to investigate whether AP pretreatment induces cell conditions that influence the inflammatory response (Fig. 2b).

In the first experiment, stimulation of Caco-2 cells with AP resulted in decreased expression of CXCL1 in TNFα-treated cells and DEFB1 in TNFα-treated and untreated cells; expression of IL-8 was increased in TNFα-treated cells, expression of GPX2 was unaffected (Fig. 2a).

After cells were pretreated with AP, stimulation of Caco-2 cells with AP still resulted in decreased expression of CXCL1 in TNFα-treated cells and DEFB1 in TNFα-treated and untreated cells. Expression of GPX2 was increased in TNFα-treated cells, expression of IL-8 was unaffected (Fig. 2b).

### Performance parameters and selected clinical parameters of AP-treated piglets

**Fig. 3:**
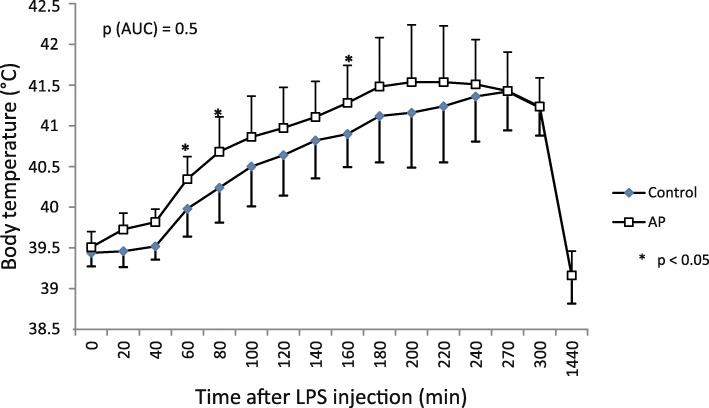
Body temperature of piglets challenged with lipopolysaccharides (LPS). Body temperature (°C) of piglets challenged with lipopolysaccharides (LPS) at a dose of 25 μg per kg body weight i.p. Three weeks prior to the LPS treatment, the groups received a diet with *Agrimonia procera* powder (10 g/kg diet) or a diet without *Agrimonia procera* (control). Data represent the mean values and SD (*n* = 11 for the *Agrimonia procera* group; *n* = 5 for the control group). AUC = area under the curve. * *p* < 0.05

**Fig. 4:**
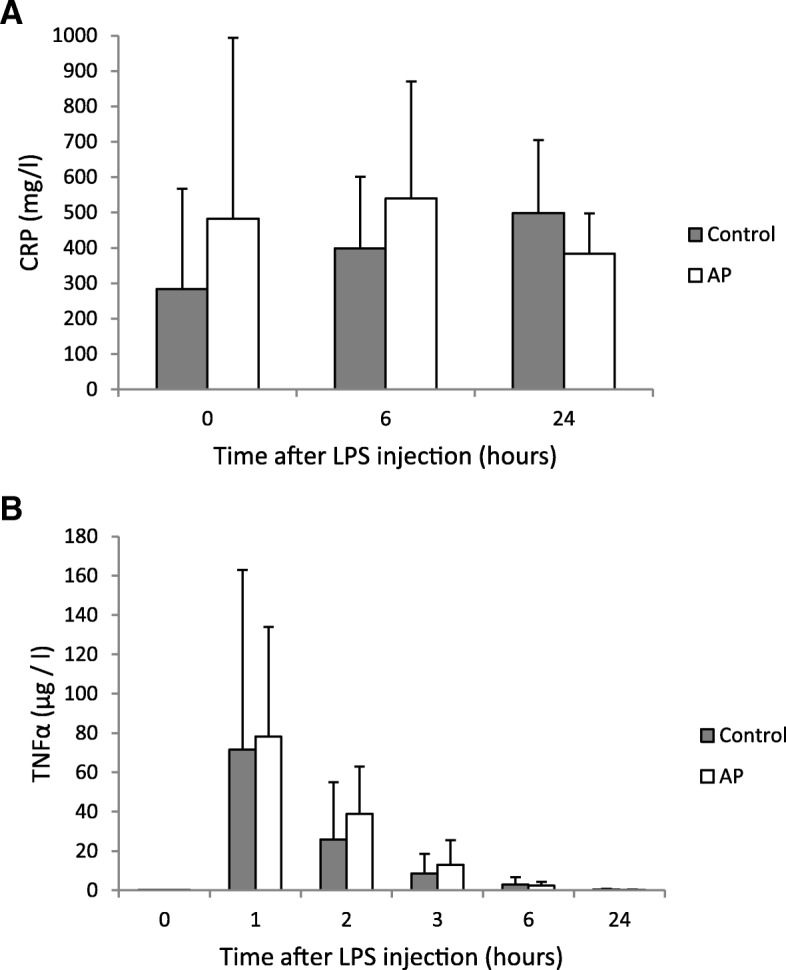
Concentrations of CRP (**a**) and TNFα (**b**) in the plasma of piglets after LPS injection. Concentrations of CRP (**a**) and TNFα (**b**) in the plasma of piglets at different points in time after a single LPS injection (25 μg/kg body weight i.p.). Piglets were fed diets without (control) or with *Agrimonia procera* (AP, 10 g/kg diet) for 3 weeks before the challenge. Data represent the mean values and SD (*n* = 12 for the *Agrimonia procera* group; *n* = 5 for the control group)

In experiment 1, the single LPS injection induced a rise in body temperature by more than 2 °C in both groups of piglets (Fig. 3). Piglets pretreated with AP prior to the LPS application showed a faster and a stronger increase in body temperature at some points in time than the control piglets (Fig. 3). However, AUC showed no difference between the two treatment groups (*p* = 0.5). Approximately 4.5 h after LPS injection, the body temperature of piglets started to decline in both groups. Analysis of plasma CRP before LPS injection and 6 and 24 h afterward revealed no difference between the two groups of piglets and no differences between the three analyzed points in time (Fig. 4a). Plasma TNFα concentration was markedly increased 1 h after LPS injection and declined afterwards, reaching its basal level 24 h after LPS injection (Fig. 4b). However, there were no differences in plasma TNFα concentration between the two groups of piglets.

In experiment 2, food intake, final body weight, daily body weight gain and food conversion ratio did not differ among the three groups of piglets (Table 2). Likewise, no impact of AP was found on growth performance after the 23-day post-intervention period (Table 2). During the intervention period, the calculated AP intake per piglet was 1.1 g/d in the AP1 group and 11.5 g/d in the AP2 group. Analysis of feces after the intervention period at day 15 revealed that piglets treated with AP tended to have higher fecal dry matter than controls (*P* = 0.055). Correspondingly, the number of piglets with fecal dry matter < 15% was reduced in both AP groups compared to the control group (Table 2). The total antioxidant capacity of plasma, expressed as TEAC and assessed after the intervention period, tended to be higher in piglets treated with AP than in control piglets (*P* = 0.087, Table 2).

**Table 2:** Growth performance and health parameters of piglets fed *Agrimonia procera* compared to control piglets

Parameters	Control (0 g/l)	*Agrimonia procera* (AP)	
		AP1 (1 g/l)	AP2 (10 g/l)
Mean feed intake (g/d)^a^			
Intervention period (days 1–15)	220 ± 28	199 ± 13	239 ± 37
Follow-up period (days 15–38)	814 ± 91	810 ± 16	860 ± 2
Body weight (kg)			
End of intervention period (day 15)	10.9 ± 2.3	10.7 ± 1.8	11.0 ± 1.8
End of follow-up period (day 38)	23.7 ± 3.2	23.4 ± 3.3	24.4 ± 2.6
Daily weight gain (g/d)			
Intervention period (days 1–15)	157 ± 77	155 ± 64	175 ± 76
Follow-up period (days 15–38)	550 ± 59	554 ± 85	566 ± 72
Feed conversion ratio^a^			
Intervention period (days 1–15)	1.42 ± 0.03	1.29 ± 0.15	1.37 ± 0.12
Follow-up period (days 15–38)	1.48 ± 0.13	1.46 ± 0.02	1.52 ± 0.01
Fecal dry matter content (%) at day 15	19.8 ± 8.1	22.2 ± 6.1	24.8 ± 3.4
Number of piglets with fecal dry matter			
< 15% at day 15	6	3	1
Plasma TEAC^b^ (μmol/ml) at day 15	6.28 ± 0.86	6.53 ± 0.97	7.22 ± 0.86

Growth performance and health parameters of piglets fed *Agrimonia procera* compared to controls for 15 days with a follow-up period of 23 days. Data are means and standard deviations (*n* = 20)

^a^Means of 2 piglets per pen

^b^*TEAC* Trolox equivalent antioxidant capacity

## Discussion

The aim of the study was to elucidate the antimicrobial and antioxidant properties as well as the immunomodulatory effects of AP in vivo and in vitro.

The growth rates of various bacterial strains, especially *Pediococcus pentosaceus* and all tested *E. coli* strains, were reduced by AP in vitro (Fig. 1). This is of special interest, as, during the first few weeks after weaning, most diseases (such as diarrhea) are caused by *E. coli* [19, 20]. *E. coli* colonizes the small intestine and produces toxins that impair intestinal barrier function, stimulate intestinal water influx [21] and reduce the growth of beneficial bacteria [22, 23]. Bacterial diarrhea also increases the release of reactive oxygen species in immune cells [24]. The performance-enhancing effect of phytogenic additives in pigs [25] and poultry [26] is caused by a stabilization of the intestinal flora and thus constitutes even more than a direct antimicrobial effect. Oligomeric polyphenols (such as agrimoniin) transit the gastrointestinal tract without being altered and are only marginally transported across the intestinal epithelium [27–29]. Accordingly, polyphenols have a direct effect on harmful and toxic bacteria such as various *Salmonella* and *E. coli* species. Furthermore, polyphenols reaching the colon are extensively metabolized by the microflora into a wide range of low-molecular-weight phenolic acids. Accordingly, polyphenols may have a favorable effect on the gut microflora, as shown in animals [30, 31] and humans [32]. For example, polyphenols from black tea have been shown to reduce the prevalence of diarrhea by up to 20% in piglets orally infected with *E. coli* [33]. Black tea extract has been shown to improve net fluid absorption after infection and has an inhibitory capacity towards enterotoxigenic pathogens and their enterotoxins [34]. The literature presents various explanations for the antidiarrheal effect of AP (e.g.*,* [35, 36]). First, the phenolic compound could bind to iron, thereby rendering the mineral unavailable to *E. coli* and consequently delaying its growth. Second, the potential formation of complexes with bacterial cell membrane proteins or polysaccharides could disrupt cell membrane integrity and growth. Another possibility is that AP phenolic compounds bind to enterotoxin and thereby inhibit enterotoxin internalization. As agrimoniin also showed potent inhibitory effects on 32 tested *Helicobacter pylori* strains [37] and *Staphylococcus aureus* [38], the antimicrobial efficacy of AP seems to have a broad spectrum.

In the next step, we analyzed the impact of AP on the expression of relevant defense proteins in TNFα-treated and untreated enterocytes in vitro using Caco-2 cells. Caco-2 cells are accepted as in vitro model of the intestinal epithelium and are therefore suitable to study the anti-inflammatory potential of AP. Moreover, Caco-2 cells and IPEC-J2 intestinal porcine epithelial cells show equal responses to inflammation and the added ingredients [39].

In the current study, stimulation of Caco-2 cells with AP resulted in decreased expression of CXCL1 in TNFα-treated cells.

Generally, polyphenols are able to modulate NF-κB and mitogen-activated protein kinase activation after oral administration [40]. Agrimoniin has been shown to inhibit TNFα-induced NF-κB driven transcription and nuclear translocation in a concentration-dependent manner in human gastric epithelial cells [41]. Presumably, certain oligomeric polyphenols induce anti-inflammatory effects in Caco-2 cells via direct interaction with TNFα receptors or via interactions with membrane lipids, leading to changes in the physical properties of the membrane that affect the affinity of the receptor for its ligand [42, 43]. The current in vitro experiment shows that cells treated simultaneously with TNFα and AP are characterized by increased expression of the proinflammatory cytokine IL-8. This is an unexpected finding, as polyphenols are usually described as anti-inflammatory molecules [41, 44]. On the other hand, an effective immune response to bacteria requires the activation of NF-κB to produce cytokines.

Expression of DEFB1 was significantly lower in cells treated with AP than in cells without AP treatment, independent of the presence of TNFα. Defensins, typically termed antimicrobial peptides, are important components of host defense through innate immunity. Therefore, defensins are promising candidates for use as antibiotics [45]. In addition, defensins affect the activation of NF-κB [46].

AP also had significant effects on the mRNA expression of GPX2 in cells pretreated with AP. TNFα-challenged cells treated with AP had significantly higher GPX2 expression than cells without AP treatment. GPX2 was identified to be involved in the activation of the endogenous antioxidant defense system in Caco-2 cells [47] and is a target gene of Nrf2. Certain isolated phytochemicals inhibit the NF-κB pathway and simultaneously activate the Nrf2 pathway [10]. Moreover, there is a significant correlation between TEAC and estimated phenolic content. This anti-inflammatory activity suggests a contributory role of polyphenols in the anti-inflammatory activity of several culinary herbs that have been investigated [44].

Most gram-negative bacteria, such as *E. coli*, have LPS as an intrinsic component of the outer membrane. This component activates the immune system and stimulates the expression of proinflammatory cytokines, such as TNFα [48]. Therefore, the anti-inflammatory potential of AP was investigated in vivo in LPS-challenged animals.

Application of LPS induced significantly elevated body temperatures in animals. The AUC of body temperature showed no difference between the treatment and control groups, whereas piglets pretreated with AP prior to LPS application showed faster and larger increases in body temperature than control piglets at some points in time (Fig. 3). In addition, piglets pretreated with AP appeared to release more TNFα than controls (Fig. 4). Although treatment with LPS does not completely mimic the physiological effects of infection and inflammation in commercial practice [49, 50] as, e.g.*,* LPS and living *E. coli* induce different immunological profiles in weaned pigs [51], previous studies advanced the concept that pigs injected with LPS could imitate the response of piglets exposed to microorganisms in a conventional environment [52, 53]. In pigs, LPS evokes a rapid febrile response, reduces feed intake and increases plasma TNFα [54, 55]. Fever decreases the toxicity and growth of bacteria, boosts the immune system, inhibits LPS formation and increases survival rates [56–58]. Herbal extracts can stimulate the immune system [59, 60]. After 5 weeks of dietary supplementation with polyphenol-rich cereals, leukocyte functions were improved [61]. Intraperitoneal injection of agrimoniin increased the number of peripheral white blood cells and the ratio of monocytes in rodents [62], and *A. eupatoria* tea consumption led to decreased interleukin 6 levels in humans [14]. Moreover, agrimoniin-containing *Potentilla erecta* showed significant erythema-reducing activity in vivo [63].

As different herbs were also shown to improve growth performance parameters in piglets [64, 65], we analyzed the impact of AP on growth performance.

AP treatment had no impact on the analyzed growth performance parameters in piglets (Table 3). Only fecal dry matter and total plasma antioxidant capacity tended to be higher in piglets treated with AP than in control piglets (Table 3). Additionally, *A. eupatoria* L. failed to affect the growth performance of farm animals [66]. In contrast, AP had a positive effect on nitrogen retention and food conversion ratio [18]. Antioxidant effects were also found in other studies [67]. The inconsistent results could be explained by variation in the composition of phytobiotics. The potency of medical plants depends on factors such as growing location, harvest conditions, extraction and stabilization methods, and storage conditions [68].

**Table 3:** Characteristics of primer sequences used for quantitative real-time RT-PCR analysis

Gene	Forward primer (from 5′ to 3′) Reverse primer (from 5′ to 3′)	Product size (bp)	NCBI GenBank number
CXCL1	ATGCTGAACAGTGACAAATC	96	NM_001511.3
	TCTTCTGTTCCTATAAGGGC		
IL-8	GTTTTTGAAGAGGGCTGAG	89	NM_000584.4
	TTTGCTTGAAGTTTCACTGG		
GPX2	AATTTGGACATCAGAACTGC	190	NM_001115136.1
	GGCTGCTCTTCAAGATTTAG		
DEFB1	AGGTGGTAACTTTCTCACAG	192	NM_005218.3
	AAGTTCATTTCACTTCTGCG		
GAPDH	GACCACAGTCCATGCCATCAC	453	NM_002046.5
	TCCACCACCCTGTTGCTGTAG		
RPLP0	TCGACAATGGCAGCATCTAC	223	NM_001002.3
	GCCTTGACCTTTTCAGCAAG		

*CXCL1* Chemokine (C-X-C motif) ligand 1, *IL-8* Interleukin-8, *GPX2* Glutathione peroxidase 2, *DEFB1* Beta-defensin 1, *GAPDH* Glyceraldehyde 3-phosphate dehydrogenase, *RPLP0* Ribosomal phosphoprotein P0

## Conclusions

The current studies showed that AP exerted antimicrobial effects in vitro, as the growth rate of various bacteria strains was negatively affected. Interestingly, the additive showed inflammatory potential in vitro and increased the immune response in LPS-treated animals*.* In addition, AP appeared to exert antioxidative effects in vivo. As an effective immune response to bacteria requires the activation of NF-κB to produce cytokines, AP has the future potential to function as an effective alternative to antibiotics in animal husbandry. Therefore, prospective studies should focus on animal experiments showing that AP-pretreated animals are in fact able to respond more rapidly and appropriately to a bacterial infection than non-treated control animals.

## Methods

### Characterization of secondary plant compounds in *Agrimonia procera*

Flavonoids and agrimoniin were extracted from AP (aerial parts, harvested in 2011 in Zappendorf and milled to pass through a 1 mm sieve) with ethanol (50%) in an ultrasonic bath for 10 min at 30 °C. After centrifugation at 450 rpm for 10 min, the supernatant was analyzed by HPLC using an Agilent 1100 system (Agilent, Santa Clara, USA) equipped with an UV detector. For flavonoid analysis, a reversed-phase column (Kinetex®, 5 μm C18, 150 × 4.6 mm^2^) and a guard column (Security Guard Ultra, both Phenomenex, Aschaffenburg, Germany) were used. The mobile phase consisted of (A) water + 0.1% trifluoracetic acid and (B) acetonitrile + 0.1% trifluoracetic acid (time table: from A/B 90:10 to 60:40 in 30 min). For agrimoniin analysis, a reversed-phase column (Luna®, 3 μm PEP(2), 150 × 4.6 mm^2^) and a C18 guard column (both Phenomenex) were used. The mobile phase consisted of (A) water:methanol:formic acid (95:6:0.1, *v*/*v*/v) and (B) methanol:formic acid (100:0.1, v/v) (time table: from A/B 95:5 to 5:95 in 38 min). In both analyses, the flow rate was 1 ml/min and the injection volume was 5 μl. P-aminobenzoic acid was used as an internal standard.

### Antimicrobial efficacy test of *Agrimonia procera*

The potential antibacterial effects of AP were elucidated by treating *Escherichia (E.) coli* strains (DSM 1103, DSM 6895, DSM 8703), *Salmonella* (*S.*) *enterica* ssp*. enterica* serotype Typhimurium (ATCC 13311) and the lactic acid bacteria *Lactobacillus (L.) casei* (DSM 20011) and *Pediococcus pentosaceus* (DSM 20336) with an aqueous extract of AP. The strains were obtained from German Culture Collection (DSMZ, Braunschweig, Germany). To this end, bacterial strains were initially cultivated in LB medium (Carl Roth, Karlsruhe, Germany) for *E. coli*, CASO-Bouillon (Carl Roth) for *S. enterica* ssp. *enterica* ser. Typhimurium*,* and MRS medium (Carl Roth) for *L. casei* and *Pediococcus pentosaceus* at 37 °C on a shaker at 150 rpm (Infors, Bottmingen, Schweiz)*.* To produce the aqueous extract of AP, we infused 1.5 g of AP powder (Exsemine GmbH, Salzatal, Germany) in 150 ml of boiling water for 30 min. Aliquots of the aqueous extract were then added to the respective media to yield a concentration of 1 mg of AP aqueous extract per ml. After cooling, the test media were filtrated with a 0.2 μm syringe filter under sterile conditions and inoculated with the bacteria strains. The growth of bacteria was estimated by measuring the optical density at 600 nm over a period of up to 28 h. Due to the different growth rates of the bacterial strains, the investigation periods for the response of bacterial strains to AP varied. The control groups used the same media without AP.

### In vitro experiments with Caco-2 cells

To elucidate the effects of agrimoniin on mRNA expression of inflammatory molecules and host defense peptides in enterocytes, we employed the human colon carcinoma cell line Caco-2. Cells were grown in MEM (Life Technologies, Darmstadt, Germany) supplemented with 10% fetal bovine serum (FBS, Life Technologies), 1% nonessential amino acids (PAA, Pasching, Austria) and 0.5% gentamicin (Life Technologies). Cells were maintained at 37 °C in a humidified atmosphere with 5% CO_2_. The medium was changed every 2 days. The cells were cultivated in culture flasks (Greiner, Frickenhausen, Germany) and passaged regularly before reaching confluence. For treatment, cells were seeded in 24-multiwell plates (Greiner) at a density of 1 × 10^5^ cells per ml of medium per well, cultured to confluence, and then cultured for an additional 6 days.

Two cell experiments were conducted, both employing a two-factor design with the treatment factors agrimoniin and TNFα. In the first experiment, cells were simultaneously treated with 0 or 10 ng/ml TNFα (R&D Systems, Abingdon, UK) and 0 or 50 μM agrimoniin for 1, 6 and 24 h.

The second experiment aimed to investigate whether agrimoniin pretreatment induces cell conditions that modulate their inflammatory response. To this end, cells were preincubated with 0 and 50 μM agrimoniin for 4 h. Thereafter, the preincubation medium was removed and replaced with fresh medium containing 0 or 10 ng/ml TNFα and 0 or 50 μM agrimoniin for 1, 6 and 24 h. The AP powder used for both experiments was dissolved in DMSO (Sigma), TNFα was dissolved in PBS. Cells treated with TNFα- and agrimoniin-free medium made up the control groups. The media for the controls contained corresponding volumes of DMSO (< 0.1%) and PBS in place of TNFα and agrimoniin, respectively.

At the end of each experiment, the supernatants were removed, and the cells were frozen and stored at − 80 °C until analysis of mRNA expression of chemokine (C-X-C motif) ligand (CXCL1), interleukin-8 (IL-8), glutathione peroxidase 2 (GPX2), and beta-defensin 1 (DEFB1). Each experiment was repeated 3 times.

### Real-time RT-PCR analysis

Total RNA was isolated from Caco-2 cells using peqGOLD TriFast™ (Peqlab, Erlangen, Germany) according to the manufacturer’s protocol. RNA concentration and purity were determined by optical density at 260 and 280 nm, respectively, using a spectrophotometer. Total RNA (1.2 μg) was subjected to first-strand cDNA synthesis at 42 °C for 60 min using M-MuLV RT (Thermo Fisher Scientific Inc., Waltham, MA, USA) and oligo dT18-primer (Operon Biotechnologies, Cologne, Germany). The mRNA concentrations of reference and target genes were measured with real-time detection PCR using SYBR® Green I (Sigma-Aldrich GmbH) and the Rotor-Gene 2000 system (Corbett Research, Mortlake, Australia) to determine the relative mRNA concentrations of the target genes. PCR was performed with 0.5 U of GoTaq Flexi DNA polymerase (Promega, Mannheim, Germany), 200 μM dNTP (Ares Bioscience, Cologne, Germany) and 26.7 pmol of the specific primers (Operon Biotechnologies). For determination of relative mRNA expression, a threshold cycle (C_t_) was obtained from each amplification curve using the software Rotor-Gene 4.6 (Corbett Research). The housekeeping genes glyceraldehyde 3-phosphate dehydrogenase (GAPDH) and ribosomal phosphoprotein P0 (RPLP0) were applied for normalization; both showed high stability. Relative mRNA concentration was calculated according to Pfaffl [69]. The amplification and specificity of PCR products were controlled with agarose gel electrophoresis. The characteristics of the primers used are shown in Table 3.

### Performance and selected clinical parameters of AP-treated piglets

Animal experiments were conducted using weaned hybrid piglets [(German Landrace × German Edelschwein) × Pietrain]. All animals were kept in an environmentally controlled facility with light from 6:00 am to 6:00 pm, relative humidity between 55 and 60%, and a temperature of 29 °C at the beginning, which was decreased gradually to 22 °C at the end of the experiments. Both studies were approved by the local Animal Care and Use Committee of the council of Saxony-Anhalt (Landesverwaltungsamt Sachsen-Anhalt, Germany; approval number of the first animal trial: 42502–2-1143MLU; approval number of the second animal trial: 42502–3-716MLU).

The first experiment was conducted to elucidate the response of LPS-challenged piglets to AP treatment. Twelve castrated 7-week-old male piglets were assigned to 2 groups of 6 animals each. The piglets were housed individually in cages and received a basal diet that contained the following (per kg): 380 g of wheat, 195 g of barley, 180 g of soybean meal, 100 g of corn, 50 g of whey powder, 30 g of wheat bran, 25 g of soybean oil, and 40 g of standard premix to meet the nutrient requirements for piglets [70] supplemented with either 0 g (control) or 10 g of AP powder (dried and ground aerial parts of AP) per kg of diet for 3 weeks. After this 3-week-period, the piglets were challenged with a single LPS treatment. Specifically, 25 μg of LPS per kg of body weight was injected intraperitoneally after the diets were delivered at 8:00 a.m. LPS (from *E. coli* 0111:B4, Sigma-Aldrich L-2630, Munich, Germany) was dissolved at 250 μg/ml in physiological saline solution. On the day of injection, the piglets had a mean body weight of 15.7 kg. After LPS injection, the piglets were clinically monitored for a period of 24 h. Rectal temperature was measured with a digital thermometer every 20 min during the first 5 h and at the end of 24 h. Blood samples were collected from the jugular vein immediately prior to LPS administration (0 h) and 1, 2, 3, 6, and 24 h afterwards to analyze the plasma concentrations of C-reactive protein (CRP) and TNFα.

In the second experiment, we aimed to investigate the effects of AP in apparently healthy piglets. For that purpose, sixty castrated 4-week-old male and female (1:1) piglets with a mean body weight of 8.5 kg were randomly assigned to 3 groups of 20 animals each (10 male and 10 female). Two groups received AP as an aqueous extract containing 1 g or 10 g of AP per liter for 15 days. The third group received no AP extract and served as a control. All groups received the basal diet mentioned above. The aqueous AP extract was prepared by infusing dried AP (Exsemine GmbH, Salzatal, Germany) in hot water for 10 min with subsequent filtration. The piglets were housed in flat-deck pens (10 piglets per pen) and had free access to food and water. Blood samples were drawn from the jugular vein to analyze antioxidant activity in plasma at the beginning and the end of the experiment. Feces samples were collected and assessed for their dry matter content (on day 15). The intake of food and aqueous AP extract was recorded daily; body weights were recorded weekly. The performance data were also assessed for a postexperimental period of 23 days.

### Analysis of plasma TNFα, CRP and antioxidant capacity

TNFα and CRP concentrations in plasma were determined by using an ELISA kit (R&D systems, Minneapolis, USA). The antioxidant capacity of plasma was assessed by using a Trolox equivalent antioxidant capacity (TEAC) assay as described elsewhere [18].

### Statistical analysis

Data collected from Caco-2 cell experiments were analyzed with two-way ANOVA. The effects of the two factors agrimoniin (0 vs. 50 μM) and TNFα (0 vs. 10 ng/ml) as well as their interaction were investigated. When two-way ANOVA revealed a significant effect, a post hoc comparison was performed. In the case of variance homogeneity, the means of the four treatment groups were compared by Fisher’s exact test. In the case of variance heterogeneity, the Games-Howell test was applied. Means were considered significantly different at *P* < 0.05. In the first animal experiment, the means of the two groups were compared by Student’s t-test. For body temperature, the area under the curve (AUC) was calculated for each piglet, and treatment groups were compared by means of Student’s t-test. Data obtained from the second animal experiment were analyzed by one-way ANOVA (Minitab, Version 13, State College, PA, USA). In the case of significant F-values (P < 0.05), means were compared by the Fisher’s least significant difference (LSD) test.

## Abbreviations

AP: *Agrimonia procera*
AUC: Area under the curve
CXCL1: Chemokine (C-X-C motif) ligand 1
DEFB1: Beta-defensin 1
GPX2: Glutathione peroxidase 2
IL-8: Interleukin-8
LPS: Lipopolysaccharide
NF-κB: Nuclear factor-kappa B
TNF: Tumor necrosis factor
